# Fairness in Assessment: Identifying a Complex Adaptive System

**DOI:** 10.5334/pme.993

**Published:** 2023-07-28

**Authors:** Nyoli Valentine, Steven J. Durning, Ernst Michael Shanahan, Lambert Schuwirth

**Affiliations:** 1 Prideaux Discipline of Clinical Education, Flinders University, Bedford Park, South Australia, Australia; 2 Department of Medicine, Director, Center for Health Professions Education, Uniformed Services University of the Health Sciences, Bethesda, MD, United States; 3 Flinders University, Bedford Park, South Australia, Australia

## Abstract

**Introduction:**

Assessment design in health professions education is continuously evolving. There is an increasing desire to better embrace human judgement in assessment. Thus, it is essential to understand what makes this judgement fair. This study builds upon existing literature by studying how assessment leaders conceptualise the characteristics of fair judgement.

**Methods:**

Sixteen assessment leaders from 15 medical schools in Australia and New Zealand participated in online focus groups. Data collection and analysis occurred concurrently and iteratively. We used the constant comparison method to identify themes and build on an existing conceptual model of fair judgement in assessment.

**Results:**

Fairness is a multi-dimensional construct with components at environment, system and individual levels. Components influencing fairness include articulated and agreed learning outcomes relating to the needs of society, a culture which allows for learner support, stakeholder agency and learning (environmental level), collection, interpretation and combination of evidence, procedural strategies (system level) and appropriate individual assessments and assessor expertise and agility (individual level).

**Discussion:**

We observed that within the data at fractal, that is an infinite pattern repeating at different scales, could be seen suggesting fair judgement should be considered a complex adaptive system. Within complex adaptive systems, it is primarily the interaction between the entities which influences the outcome it produces, not simply the components themselves. Viewing fairness in assessment through a lens of complexity rather than as a linear, causal model has significant implications for how we design assessment programs and seek to utilise human judgement in assessment.

## Introduction

Assessment design in health professions education is continuously evolving in response to new insights, ideas and research findings. Historically, assessment has been seen mainly as a measurement problem, with reliability and validity being key components of assessment [1]. Over time, however, evolving views about learning and rater cognition, shifting social ideals and understandings of the limitations of high stakes tests has challenged the idea that objectivity is the gold-standard of assessment [2,3,4,5,6,7,8,9,10].

As a result, there has been an increasing push to better utilise the role of human judgement in assessment [2,3,4,5,6,7,8,9,11]. This was initially was under the guise of ‘reliable subjectivity’, utilising assessor training and large samples to ensure sufficient reliability of the assessment [12]. But more recently it has been acknowledged that rater variance may provide meaningful idiosyncrasy and should be embraced rather than controlled [3,6,13,14,15].

However, assessment still needs to be fair. Subjective human judgements do not add meaningful idiosyncrasy if they are unfair to either learners or society. Nor will fair judgements add meaning if they are part of unfair assessment systems. So, addressing what makes human judgement fair in health professions assessment is essential in legitimising subjective judgements in our assessment programs.

Fairness is often implied in assessment programs, but is not usually explicitly articulated as there is no simple definition for this complex construct [16], and fairness is dependent on cultural beliefs, social contexts and practices [10]. Despite the lack of explicit definition, the underpinnings and constituents of fairness are implied in the medical education and broader education literature. A literature review brought these inferences and underpinnings together to create a theoretically constructed conceptual model [16]. This literature review noted that the multifaceted construct of fair human judgement could be conceptualised through values, which are upheld at an individual and system level [16]. A further study exploring the understanding of residents’ and supervisors’ perspectives of fairness built on the theory-derived conceptual model, demonstrating that the components of fairness could be explicitly articulated whilst still embracing the complexity and contextual nature of health professions assessment [17]. This study noted that at an individual level, contextual, longitudinally-collected evidence, which is supported by narrative, and falls within ill-defined boundaries is essential for fair judgement decisions. Assessor agility and expertise are needed to interpret and interrogate this evidence, help identify fuzzy boundaries and provide narrative feedback to ensure learners can improve. At a system level, factors such as multiple opportunities for learners to demonstrate competence and improvement, multiple assessors to allow for different perspectives to be collected and triangulated, and documentation are all needed for fair judgement. These system features are supported through the concept of procedural fairness which provides transparent expectations, allows for fit-for-purpose, individualised, proportional judgements, and supports dialogue and engagement with the learner. Finally, the environment in which the assessment decisions are made needs to be considered for fair judgments [17]. The resulting model can assist in developing narratives to ‘negotiate’ fairness between stakeholders.

Whilst this was helpful, given the fundamental nature of fairness in assessment, it is important to understand stakeholder perspectives, such as expert assessment leaders. Their insights could further help translate this concept of fairness and bring change to educational practice. In this study we, therefore, addressed the following research aims:

1. To understand what the characteristics of fair judgement are from assessment leaders’ perspectives.
2. To compare and contrast these understandings with our previously reported theoretically constructed conceptual model [16,17].
3. To understand how these understandings and theoretical aspects translate to practice and suggest design principles to assist in the practical application of a theory derived conceptual model.

## Methods

### Reflexivity

We took a subjectivist, inductive approach to this research, assuming that fairness as a reality is socially constructed, and that individuals and social groups share interpretations and understandings of the reality of fairness [18]. The components of fair judgement in assessment are constructed by individuals and institutions, and change over time and across cultures. Therefore, we also took a constructivist stance in that the meaning of fair judgement is constructed by stakeholders, rather than the idea that there is a simple, universal true definition of fairness. Collecting data from multiple perspectives will therefore assist in gaining a richer and more nuanced understanding of this phenomenon [18].

Reflexivity was employed throughout the research process and is described through the dimensions of ‘personal’, ‘interpersonal’, ‘methodological’ and ‘contextual’ [19]. The research team consists of experienced HPE researchers and clinicians, all familiar with the study content, having undertaken previous studies on fairness in assessment. The research team members work in diverse contexts, representing a range of specialties and HPE research environments across different continents. All team members consider themselves to be social constructivists. LS, NV & MS have been previously involved in medical education in Australia. The diversity of experiences of the research team was leveraged allowing for a range of perspectives enabling rich team discussions during data interpretation [20]. NV’s interest in fairness initially stemmed from her role in medical education and as a senior clinician. However, her perspective has shifted slightly as she has now recommenced as a trainee in a different medical specialty. NV approaches fairness from the dual perspective of both a PhD candidate and a clinician. SJD is interested in fairness as a director of academic programs spanning the continuum. SJD believes that nonlinearity and complexity often shape our interactions. SJD approached the topic and findings as both a PhD scholar and practicing physician. EMS is a full-time practicing clinician with a lifetime career committed to medical education. His interest in fairness has developed through his work as a program director and educator of students and physician trainees. LS has an interest in fairness as a researcher in assessment and with an interest of understanding assessment in a post-psychometric era. He believes that nonlinearity and complexity often shape our interactions. LS approached the topic and findings as both a research scholar and a professor of medical education. He is the first in his extended family to attend college and therefore, fairness is an important value for him.

### Participants

Eligible participants were assessment leaders from the 23 medical schools in Australia and New Zealand. All 29 members of the assessment leads of the Medical Deans of Australia and New Zealand were invited to participate in 90-minute focus group conducted via Zoom. We chose focus groups to allow individuals to build on other group members’ responses, allowing for dynamic interactions [21]. As an aim of the study was to understand how previously identified theoretical aspects translated to practice, participants were asked to design an assessment program for a fictional medical school utilising subjective judgements while trying to make these fair to both learners and society. Participants were instructed to employ blue sky thinking; we posed no barriers to time, money or supervisor engagement as this was not the aim of the study. A collaborative white board, Miro, was used to facilitate discussions. We provided no incentive to participate. Ethics approval was obtained (Flinders University: 4297).

### Analysis

Data collection was undertaken from July to September 2021. NV conducted the focus groups and had limited familiarity with the participants. Focus group were recorded and transcribed verbatim without identifying data. Focus groups notes and the shared white board were included in data analysis. NVivo, a qualitative analysis software, was used to assist with data management.

Collection, analysis and coding of the data occurred simultaneously, each informing the other. NV initially read each transcript line-by-line to allow for familiarisation with the data. The analysis process involved discussions between researchers and comparison of different codes between and within transcripts to clarify, confirm and categorise codes. After focus groups and initial data analysis was complete, we reviewed our data in light of the previous conceptual model, examining how these findings elaborated or contradicted the previous findings [17].

## Results

Of the 29 invited assessment leaders, 19 volunteered to participate but three withdrew prior to the focus groups. The five focus groups were attended by 12 females and four males from 15 medical schools. Fourteen medical schools were located across all six states of Australia and one was located in New Zealand. Two medical schools were located in large regional centres, 13 were in major cities. All participants had experience in assessment design and delivery at their respective medical schools. Participants’ academic titles at the time of the focus groups are listed in Table 1.

**Table 1:** Academic titles of focus group participants.


Academic Assessment Lead	Academic Lead Assessment	Acting Dean

Associate dean	Associate Dean, Learning and Teaching	Associate professor (3 participants)

Chief Examiner and Head of Assessment	Director of Assessment	Director Medical School

Discipline Leader	Doctor of Medicine Program Director	Faculty Dean

Head of Assessment		


**Figure 1:**
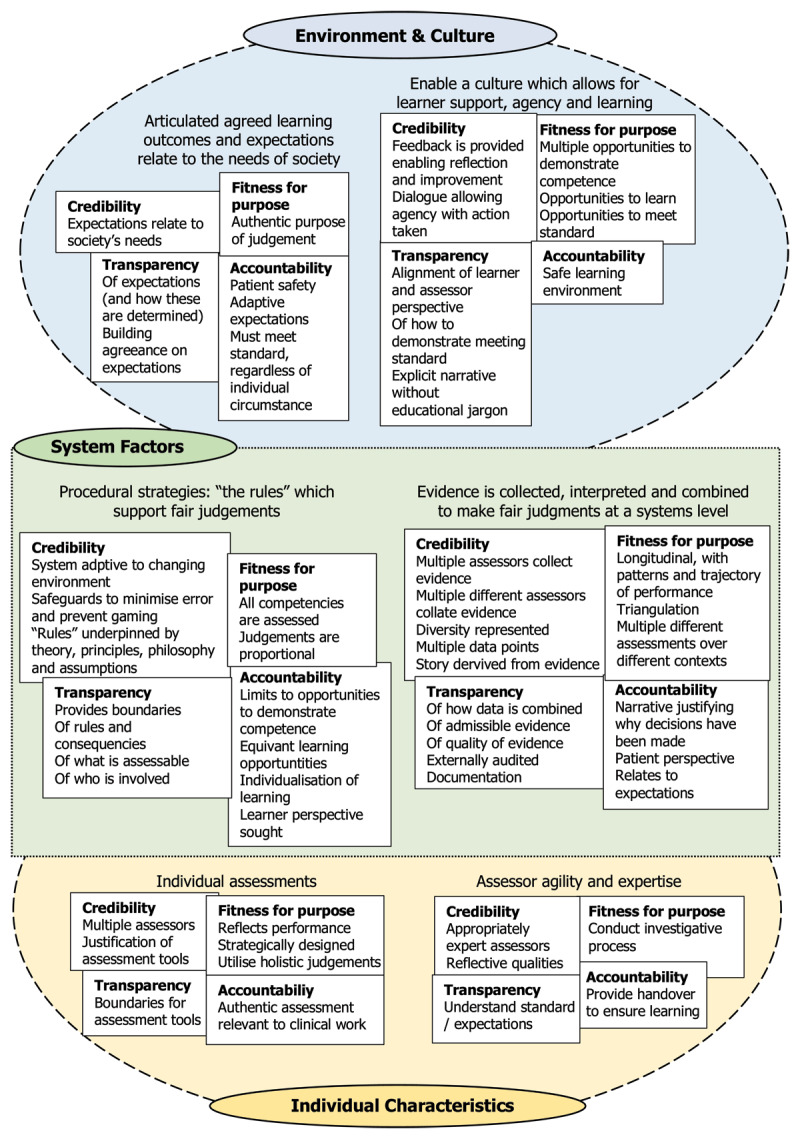
The components of fair judgement.

Fair judgements are more than just the individual judgements themselves. Judgements are not considered fair unless the environment, culture, and system in which they are made is also considered fair as demonstrated by the different sections in Figure 1. So, in evaluating fairness conceptualisations and design decisions, there are many aspects of the assessment system which need to be considered in conjunction with each other.

### Environment and Culture

Individual judgement decisions interact with their environment and culture; and so need to occur in environments and cultures which are fair to both society and learners.

#### Articulated agreed learning outcomes and expectations relate to the needs of society

One way of linking individual judgements with the environment is through transparency of expectations, typically through establishing agreement with relevant stakeholders. That way, fairness is ensured through allowing assessors and society opportunity to provide relevant perspective on competence and its practical usefulness. *“That common standard, even if it’s subjectively deployed, could be understood in some written words to be where everyone was aiming for.” P1*

Patient safety is a central concept in society’s needs and thus essential for judgements to be fair to patients. This includes ensuring that learners meet agreed expectations at certain points in time, regardless of their individual circumstances. *“if they’ve got all these extenuating circumstances et cetera but the other side of that is the duty to assure to the public that the student is competent and safe.” P9 But* these expectations are not static, and so fairness also includes adapting learning to meet society’s ongoing evolving needs.

#### Enable a culture which allows for learner support, agency, and learning

Turning to the learners themselves, fair judgements need to be accompanied by meaningful feedback that enables reflection and learning. If assessments are just summative hurdles to clear, they unfairly limit opportunities for students to learn and grow in their journey to becoming health care professionals.

Fairness also requires that the assessment and feedback are a dialogue and enable the learner to share their perspective on the judgement and take agency over their own learning, with action taken following this. “*you have to show that you’ve heard the student’s story.” P6*

Safe, fit-for-purpose learning and assessment environments are essential for fairness to the learner themselves, and are in the best interest of patient care as they allow learners to continually improve. A learner who feels safe enough to recognise their weaknesses and to focus on continual improvement is more likely to be educated as a lifelong learner, even after graduation. *“Competency isn’t a do it once pass fail. It’s, didn’t do well so have another go. Didn’t do well, have another go, more feedback, have another go, more feedback.”P8*

Transparency within the learning environment allows for reflection and learning and thus is essential for fairness. The narrative used in both expectations and feedback therefore needs to be clear, explicit and without educational jargon to allow for this learning. *“Our university in its infinite wisdom has stopped us using those sort of descriptors and they’ve told us we have to give feedback to students on their university scale … it is causing all sorts of issues” P5*

Institutions must also be transparent about how learners can demonstrate that they are meeting the expected standard and what they need to do if not. In addition, transparency ensuring the learner is aware of how they are performing against expectations is required as it allows for alignment of learner and assessor perspectives. A surprise judgement is considered unfair as it denies the learner the opportunity to improve. *“it’s the no unexpected news at the end, because they’ve been forewarned as they’ve gone through.” P1*

### System Factors

#### Procedural stategies: ‘the rules’ which support fair judgements

Procedural strategies provide boundaries at a system level. These provide clarity for assessors and protection for the learners thus allowing for development of a partnership. Procedural strategies includes ensuring transparency of rules and consequences, of what is assessable, who is involved in the assessment process and provision of safeguards to minimise sources of error and prevent gaming. *“So, what’s fair game for not being assessed, like asking a stupid question, let’s say. We’re not going to judge on that.” P6*

Procedural strategies can also facilitate fairness through ensuring appropriate proportionally is assigned to judgements. This proportionality might be aligned with the stakes of the assessment, the richness of the information from the assessment or the number of opportunities to pass an assessment.

To meet the agreed learning outcomes and society’s expectations of a practicing professional, multiple competencies are needed. Procedural strategies were suggested to ensure judgements could only be fair at a system level if all competences were likely to be assessed. *“…looking at the domains across different assessments” P2*

It was acknowledged that learners develop competence at different paces, and if the aim is to develop competent health practitioners, then it is fair to allow individual variation. *“We’re allowing some to go slower and some to go faster” P7*

However, this allowance for individualisation of learners needs to be balanced with the need to ensure fairness to society through placing limits on the opportunities provided to demonstrated competence. Furthermore, failure to fail is also unfair to learners as this may deny them the opportunity to learn and undertake remediation. *“…is not fair is that the poorer students are given many more opportunities to scrape through the course rather than [fail]” P1*

Due to the unpredictable nature of workplace-based learning and assessment, not all learners will have exactly the same experiences, but they are all entitled to the same quality of learning experiences and assessment. So, equity may be more important than standardisation.

Fairness can be supported by ensuring the “rules” are underpinned by theory, principles, philosophy and assumptions, providing a framework for the fuzzy boundaries of the procedural strategies, and a guide for future scenarios. As the environment and situations change, the system will need to be reviewed, evaluated and adapted to ensure it remains fit-for-purpose and fair to both learner and society. *“It probably is principles-based. It’s probably influenced by theory. It probably is conceptually based as well. It’s probably strategically designed. It’s probably purposeful.” P3*

Given these fuzzy boundaries, student perspective and trust in the system are essential. Trust is important in all aspects of the system, from expectations, to process, to decision making. There are several ways this trust can be built, but without it, judgements are not considered fair, as learners do not believe their interests are considered.

*“I think that was one of the best things that we did [having students on competency committees] for the student body because no matter how much work we’d done and how much communication and consultation, the thing that’s convinced them that it genuinely was low stakes was their own peers going out, going oh no, it’s true, when it goes to the panel they really look at everything together and holistically” P4*

#### Evidence is collected, interpreted and combined to make fair judgments at a systems level

At a systems level, collection, interpretation, and collation of evidence is required. To ensure fairness, a richness of data is needed to build a picture about a learner’s progress which is reassuringly comprehensive enough to make high-stakes decisions. This includes longitudinal data from multiple different assessments, over many different contexts which allows for triangulation of data and identification of patterns of performance. *“If you’re gathering narrative from a wide range of people, you’ll often start to see patterns of behaviour or a consensus appearing. That can make it more fair” P7*

Multiple longitudinal data points also allows for a trajectory to be considered reducing uncertainty about a student’s learning journery and adding to the richness of the picture.

*“…not only have you not got to the point we want you to get to, but you’re showing no inclining of making any progress either. Different story from, you haven’t quite got there but boy we’ve been really encouraged by how much progress you’ve made over the last three months and we think if you had another three months you probably will get there.”P6*

Multiple different assessors collecting evidence adds to the credibly of the picture of evidence being collated about the learner. Diversity of opinions also adds to the richness of the picture rather than creating unreliability. *“if they know … there’s going to be multiple judgements made by multiple clinicians, that multiple perspectives … then they’re much more confident in the fairness of the assessment.”P9*

Having a different group of assessors meaningfully collate and weigh up multiple pieces of evidence at a system level adds a safety net for learners and ‘on-the-ground’ supervisors alike. It ensures a second ‘check’ for learners and allows support for supervisors in decisions making, particularly for difficult decision making.

*“We always find reassuring to our supervisors that actually it’s the [university name] Board of Examiners who makes the decision. Their job is just to tell us what they saw and be as frank as they can be about the student’s performance … but we’ll take the decision-making on our shoulders, not theirs and that does help”P7*

As evidence is collated, a story is created. This story provides meaningfulness and credibility to the judgement which makes it fair. It connects the evidence with previous knowledge and experience about the learner and provides justification for the judgement, both of which help make the judgement fair. Furthermore, from the story, areas for improvement can be identified which is also essential for fair judgement.

*“So I wonder if that narrative and the pattern equals a story. … Because if you just got a six out of 10 and a seven out of 10 and a B minus, that’s not telling you a story. But a narrative – and a narrative doesn’t tell you a decision, but it contributes to a story and then once you’ve got the story, you can make a decision.”P6*

Transparency at a systems level may involve considering what evidence could be considered in fair judgements and how the quality of the evidence would be determined. Additionally, understanding how data is combined was also seen as important as it helps define assessment boundaries for the learner.

*“I think you need to be clear on how you’re adding things up too. If you’re going to say, they have lots and lots of direct observations, but actually we’re going to look at all of them at the end of the rotation and make some sort of narrative judgement based on all the feedback provided in those and you need to make that very clear for students.”P8*

External auditing of judgements can ensure accountability to learners and society and thus fairness. This requires documentation of how and why the judgement is made including the ‘story of evidence’ behind the judgement decision. This may also involve discussing the result with the learner. *“If you’ve got independent verification of the judgment, then that makes it a fair assessment in the student’s eyes type thing.”P1*

For judgements decisions to be fair, they needed to have an authentic purpose, that is to meet the needs of society, and relate back to the agreed outcomes for the learner. This provides accountability to society but also makes the judgement credible, transparent and fit-for-purpose for the learner. *“…there is a tangible outcome at the end of this which is basically work readiness” P15*

As judgement decisions relate to the needs of society, ensuring the patients’ perspective is represented is important for accountability. This may be through patient representation on committees or allowing patients opportunity to provide feedback. *“…it’s also important to give – allow some sort of patient voice in assessment as well” P15*

### Individual Factors

#### Individual assessments

To be accountable to learners, judgements are only fair if they add to the rich picture of a learners’ performance, progress, and possibly prognosis. Assessors pushing their own agenda or making judgement decisions which are irrelevant to the outcome of assisting learners to become competent healthcare providers were seen as unfair. *“the lack of relevance.. did just go off on the examiner’s flight of fantasy” P4*

This means fair judgements must only consider factors relevant to the outcome of assisting learners to become competent healthcare professionals. Any other factor, such as reputation is outside of the boundaries of fair judgement, does not add to the meaningfulness of the judgement and so therefore is unfair. *“..is this assessment an accurate … reflection of the learning outcomes or are there issues causing irrelevant ease or irrelevant difficulty to subsets of the group that we’re assessing.”P3*

To support this, individual assessments should also be transparent with boundaries for each assessment tool to ensure fair judgements.

*“…asking the right questions of the right people in the right way. Fit-for-purpose tools, these are all things that help guide and direct and support both your trainee or learner and your assessor, so they don’t go off on tangents and they know what it’s about.”P3*

In addition to multiple assessors being used at a system level to make high stakes decisions, multiple assessors can also be used at an individual assessment level. This is not from an inter-rater reliability point of view but rather to ensure that different perspectives are combined and the whole picture is seen. *“…some types of assessments actually require that type of triangulation like multisource feedback or sometimes supervised supports where you actually have to draw on the whole team”P10*

There also needs to be justification to ensure the correct assessment tool has been selected for the right situation. There is no one-size-fits-all medical school program, and credibility of the tools needs to be demonstrated to ensure the resultant judgement is also fair and fit-for-purpose. If the combination of the collected evidence is not relevant or does not add to the whole picture it leads to the perception of unfairness as it denies the learner the opportunity to be genuinely judged and provided with feedback. It also is unfair to society as the learner is denied the opportunity for improvement. *“…you need some sort of credibility with the tools. So you probably need to show that you have got the right tools out of the toolbox”P6*

#### Assessor agility and expertise

Assessors need both agility and expertise to make fair judgements. Agility is required because assessment judgements typically involve interactive processes between assessors and learners. Assessors also need to understand the outcomes and the standard to which they are assessing. Whilst diversity of perspectives adds to the richness and completeness of the picture of the learner’s progress, prejudiced perspectives due to sociocultural factors such as racism creates unfairness. Similarly, irrelevant perspectives which do not relate to the task of being a health professional also creates unfairness. *“qualities of the decision maker. What I meant by the ability to see multiple perspectives is the awareness of one’s own biases and positioning”P11*

Assessors may be required to search for extra information to make fair judgements. This is needed for saturation of information and to ensure the complete picture of a learner’s progress is known. “*I think sometimes you actually have to go back and get some additional information about some particular aspects of individual’s capacity”P14*

As previously mentioned, fair judgments necessitate meaningful feedback to be given to enable learning. This requires clinical and educational expertise and agility of assessors to ensure this is credible and fit-for-purpose. In addition, assessors can ensure their judgements demonstrate accountability to learners through providing a ‘handover’ to other assessors help facilitate future learning. *“Because if the purpose of assessment is to help medical students be good future doctors, then we would be passing on information about their strengths, and particularly about their weaknesses as they’re progressing through the course with a view of helping them, and our future patients, so that they get better doctors.”P3*

### Discussion

This study has highlighted there is no simple definition or formula for fair judgements, but rather fair judgement is multi-dimensional and context dependent. It supports the previous contextual model [17] demonstrating there are multiple layers to fair judgment; with significant overlap between these layers. The components of fairness noted in the previous study with residents and supervisors [17] were again found in this study, however, with different emphasises as this group has a different perspective, and work in different contexts.

However, perhaps more significantly, during data analysis we realised the same four components of fairness were occurring at all levels of granularity and in all contexts. We concluded we had identified a fractal. A fractal is a shape or concept, which remains the same at different scales [22]. An infinite number of repeating patterns at different sizes are combined together to give a fractal its shape. Their defining feature, is their ‘self-similarity’, that is the same shape is found regardless of whether you zoom in or out [22,23]. Our fractal pattern or ‘shape’ was made up of four components: credibility, fitness for purpose, transparency, and accountability.

Whilst our data has been presented as categories and themes, the fractals can still be seen. During our data analysis, we noted that when participants spoke about what is required for fair judgements, underlying all they said were these four elements. This occurred whether they were speaking about judgements at a ‘corridor consult’ level, at a workplace-based assessment level all the way through a competency committee meeting level. There were different emphases on these four components in different contexts and at different levels, but all four were always present. When we compared this with our previous research, these components were also noted [16,17].

A fractal is a manifestation of an underlying complex adaptative system (CAS) [24]. CAS are systems with collections of individual agents which are interconnected so that each individual agent reacts to and influences what the other agents are doing [25,26]. It is these interactions that influence the system and the emergent phenomena it produces [27,28]. Reed illustrates it as, ‘life is more than molecules and atoms – it is the complex patterns of organisation that emerge between them’ [28]. How fair judgement can be perceived as a CAS is demonstrated in Table 2.

**Table 2:** Fair judgement demonstrated as a complex adaptive system.


FEATURES OF COMPLEX ADAPTIVE SYSTEMS	AN EXAMPLE OF HOW THIS RELATES TO FAIR JUDGEMENT

	Medical schools need to determine if students meet the standard expected to graduate.

**COMPLEX** **CAS consist of individual agents [25] who make independent choices about their actions [29]. Each individual agent reacts to what the other agents are doing [28,30]. This interaction between the agents directs the CAS and influences the outcomes it produces [27,28]. The principle of connectivity is that a system’s behaviour relies less on the nature of the individual agents than on the quantity and quality of connections between them. Therefore learning how things are interconnected is often more useful than learning about the pieces [29].** **Despite the unpredictable and adapting nature of complex systems, principles and patterns arise [28]. Understanding these patterns is fundamental to understanding how the system works [26] as they guide behaviours within it [28]**.	Judgement decisions are made by a diverse group of **individuals or committees** considering multiple different **assessments** and **evidence**. Assessors are **independent** experts allowing them to make independent judgement decisions depending on their **interaction** with the data and other **individuals**. It is not possible to **create specific rules** for how judgement decisions are made. Each judgement decisions will involve different data, with different circumstances and will be perceived in different ways. Furthermore, the determination of the outcome is more than simply including more measurement points in the model. Although further data may improve judgement decisions, the **interactions** between these factors also needs to be considered. Expert assessors recognise that a **multitude of factors** should be considered in assessment and can perceive **information from multiple interactions** simultaneously process this information to **identify patterns**. Making meaning of these **relationships** is encouraged.

**ADAPTIVE** **The efficacy and effectiveness of CAS is mainly due to the adaptability of the system. Agents adapt to past experience [29,31], internal and external influences. However this also leads to unpredictability [26,28,32], and resistance to centralised control [33]. Control is dispersed; the result of a huge number of decisions made by individual agents [31].** **Work arounds and muddling through are central to CAS [29,32]. Tensions and paradox do not necessarily need to be resolved [25]. Order, innovation and progress emerge naturally from the system, they do not need to be imposed from within or from outside [32,34]. Seemingly obvious interventions can have minimal impact on system behaviour, whereas small changes can have large unintended consequences [28,30,31]**.	The assessors and the system of assessment are **adaptive**. Previous experience, new information, a different assessment method or a change in expectations causes the agents and thus the system to change. **Adaption** is often enhanced in crisis, this may be seen in the case of a struggling trainee, making decisions with incomplete data or changing environments such as pandemics. Agents **self-organise** to consciously improve the interactions between patients, learners, the environment and the university to ensure judgements are fair. The desire is often to apply more **rules**, however these rules alone are less likely to **influence** judgement decisions. If a judgement is not obvious, the system is still able to **move forward** and judgement decisions made. Effective **judgements** can emerge, even from minimum initial data. There will always be **tensions** when making judgement decisions. For example between what is fair for the patient and what is fair for the individual student, or balancing learning with assessment.

**SYSTEMS** **Complexity thinking maintains that systems can be aided by a minimal structure, such as fuzzy, ill-defined boundaries [29]. These boundaries act as constraints in that they provide a stable structure within which change can occur [26,32].** **Individual agents and CAS are embedded within wider CAS. Therefore, we cannot fully understand the individual agents or systems without reference to the others [25,33]**.	Within assessment, **boundaries, ground rules** and **processes**, can provide assessors with security and confidence to make judgement decisions. To ensure fair judgement, sufficient organisational structure is needed to keep stakeholders focused on the task, without limiting flexibility, initiative and commitment to overall improvement. Humans are not limited to one identity, but are also **members** of clinical workplaces, families and social groups which are embedded within cultural environments and wider society. These external memberships **influence** how agents behave and the perspectives they bring to judgement decisions.


These findings provide a new perspective of how fair judgement can be conceptualised in assessment. Whilst there has been an increasing push over recent years to view assessment as a system [1,13], recommendations can theoretically still be viewed from a linear, causal perspective with less consideration given to the interactions within the system, and how the system responds to these many interactions [13].

#### Implications of viewing fairness through a complexity lens

We must acknowledge though that the use of complexity science to comprehend the complex nature of medical education is not new and is indeed encouraged [26,29,30,32]. Switching focus, and taking the view of assessment as a system one step further could have significant implications. The first implication is that it is people who create the components of the fractal and their interactions, and thus it is people who create fairness. Fairness emerges from how people use and combine credibility, accountability, fitness for purpose and transparency within our assessment systems. These interactions are mediated by strategies or effectivities. Expert and agile assessors, armed with situational and contextual awareness as well as a broad repertoire of strategies navigate these components and the interactions. For example, in making a fair judgement for an end-of-term assessment, an assessor may ask other staff about a learner, obtaining multiple pieces evidence collected over time. The assessor will interact with other stakeholders, the evidence, the context and the ‘pattern’ of fair judgement. They will potentially ask other assessors to help self-calibration, and will discuss with the learner, obtaining their perspective on the assessment. Based on these interactions they will combine information in a credible way, which is accountable, transparent and fit-for-purpose to create judgement. After giving the learner the judgement, they may then adapt, perhaps by providing more targeted feedback to help the learner improve by identifying where they are not meeting expectations.

Therefore, based on our findings, fairness cannot be reduced to a linear checklist exercise, where reductionist algorithms or ‘objective’ values and methods can be used to ensure fair judgement in assessment [17]. Just as putting all of the components of a human body in a bucket does not make life, neither does simply ensuring all four fractal components of fair judgements are ticked off build fairness in assessment. In complexity, the system behaviour relies less on the nature of the individual people and strategies but more on the strength and nature of the connections between them [29]. For example, the notion of programmatic assessment contends that individual data points are insufficient to provide a fair judgement about a learner’s performance. Instead, a fair judgement requires analysing combined data, identifying factors and contexts which may influence the learner’s performance, collecting evidence to support the judgement and provide feedbacking for improvement [35,36]. Complexity thinking allows for the explicit articulation of both the components and dynamic interactions of fair judgement. Both are needed to create fairness. This has implications for the way assessments are designed and implemented.

Complexity also challenges the idea of prediction and control. In complex systems, people need sufficient freedom to interact with one another independently [25,29]. Strict rules or policies restrict the agility and freedom of people to interact with each other and if agents do not interact, fairness cannot emerge [37]. For managers and institutions, understanding how people, patterns or fractal and strategies interact is key to making changes to the direction of the CAS [38], which counterintuitively may include reducing the rules.

Despite these implications there are many unanswered questions from this research. For example, who decides what is fair and unfair and who negotiates disagreements? What happens when disagreements cannot be resolved? What happens when fairness cannot be achieved? Shared decision making with a shared narrative to negotiate fairness rather than creating rules and regulations from a top-down approach would be preferable to allow for fairness to emerge through these interactions. However, learners, assessors and intuitions may be unfair in their interactions and prevent negotiation on fairness. An external stakeholder may need to be involved in this situation to negotiate fairness. These questions highlight future areas of research. This study also focused only on the stakeholder perspective of expert assessment leaders of medical schools and did not consider the perspectives of medical students themselves. Future research should include their valuable perspective. Furthermore, given our findings, further research should now be done considering fair judgement as a CAS. For example, researchers could consider what prevents fairness from emerging, what is the influence of other systems, external powers and pressures on the dynamics of the CAS.

There are limitations to this study. Fairness is not ‘a-cultural’ and the sociocultural context in which assessment occurs is relevant [10]. Indeed what is fit for purpose, credible, accountable and transparent will be determined by the local context and culture. This study was done in a Western orientated cultural context. It is therefore plausible the findings are limited in their generalisability. In line with our ontological and epistemological views, we do not define generalisability as replicability but rather as the extent to which we have been able to incorporate sufficient different perspectives on fairness. As demonstrated by the roles held, the participants in this research were heterogeneous with different expertise and responsibility. This diversity is likely to influence their understanding of fairness.

## Conclusion

So, whilst the individual components identified in our results are not unique; approaching fairness from an ontological viewpoint of complexity is perhaps the most significant insight from this study. Within CAS, it is primarily the interaction between the entities which influences the outcome it produces, not simply the components themselves. Our study supported this premise by noting that fairness is created by people through how they use and combine the different fractal components of fairness within the assessment system. Fractal patterns can assist in enabling sense making in complex systems. Understanding fair judgement not as a linear process with a predictable trajectory but rather as a dynamic CAS may lead to purposeful, meaningful changes in our assessment systems which supports the use of fair judgement in assessment.

## Disclaimer

The views expressed herein are those of the authors and not necessarily those of the Department of Defense, Uniformed Services University of the Health Sciences or other Federal agencies.
